# Editorial: Nitrogen-cycling microorganisms under global change: Response and feedback

**DOI:** 10.3389/fmicb.2023.1166306

**Published:** 2023-03-02

**Authors:** Yongxin Lin, Hang-Wei Hu, Gui-Feng Gao, Yanjiang Cai

**Affiliations:** ^1^Fujian Provincial Key Laboratory for Subtropical Resources and Environment, Fujian Normal University, Fuzhou, China; ^2^School of Agriculture and Food, Faculty of Science, The University of Melbourne, Parkville, VIC, Australia; ^3^State Key Laboratory of Soil and Sustainable Agriculture, Institute of Soil Science, Chinese Academy of Sciences, Nanjing, China; ^4^State Key Laboratory of Subtropical Silviculture, Zhejiang A&F University, Hangzhou, China

**Keywords:** climate change, comammox *Nitrospira*, global warming, microbial community, nitrogen cycle, nutrients fertilization

Our planet is facing threats from global environmental changes, including global warming, plant invasion, atmospheric nitrogen (N) deposition and excessive nutrient fertilization, which have fundamentally altered soil biodiversity and the biogeochemical cycle of N (Zhou et al., 2020). Soil N cycle is mainly carried out by N-cycling microorganisms, which are enormously diverse and abundant, and play a critical role in soil fertility, crop productivity, water eutrophication, and nitrous oxide (N_2_O) emissions, with positive or negative feedbacks to global change factors (Kuypers et al., 2018; Wooliver et al., 2019). Over the past two decades, our understanding of N-cycling microorganisms has significantly advanced, with the discovery of many new microorganisms involving in N cycle, including ammonia oxidizing archaea (AOA), complete ammonia oxidizers (comammox *Nitrospira*), and atypical N_2_O reducers (Hallin et al., 2018; Kuypers et al., 2018). The discovery of these new microorganisms has fundamentally transformed our understanding of global N cycling, but has also increased the complexity and difficulty of studying N-cycling microorganisms (Bakken and Frostegard, 2017; Li et al., 2023; Lin et al., 2023). Despite these recent advancements in our understanding of N-cycling microorganisms, their responses and feedbacks to global change are still largely unknown. Therefore, this Research Topic focuses primarily on investigating how N-cycling microorganisms and associated N cycling processes respond to global changes. By improving our understanding of N-cycling microorganisms and their responses to global changes, we can develop better agricultural and environmental management policies to cope with future changes in global conditions (Bakken and Frostegard, 2017; Norton and Ouyang, 2019).

Unprecedented global warming is causing glacier to shrink, resulting in rising sea levels in coastal regions and an expansion of riparian wetland in alpine meadow regions, which can significantly impact N-cycling microorganisms. Chen et al. demonstrated that riparian wetland expansion increased the abundance and diversity of diazotrophs, and resulted in changes in the diazotrophic community structure as compared to alpine meadows. The assembly of diazotrophic community was primarily driven by drift and dispersal limitation. However, the expansion of riparian wetland under global warming may increase the relative importance of drift in the assembly process. Additionally, nutrient fertilization, including chemical fertilizer and manure application, can have a significant impact on the diazotrophic community (Lin et al., 2018; Dai et al., 2021). Lin et al. found that manure application increased soil aggregation and the abundance of diazotrophs, which preferred to inhabit larger sized aggregates. The abundance of diazotrophs in large macroaggregates was positively correlated with crop yield. While stochastic processes primarily controlled the assembly of diazotrophic community in the control, low-rate manure application increased the relative contributions of deterministic processes. These findings suggested that global environmental change factors, such as global warming and nutrient fertilization, have significant impacts on soil diazotrophic communities, which in turn can affect biological N fixation in terrestrial ecosystems.

Nutrient fertilization not only has a strong influence on diazotrophic communities, but also has a substantial impact on other N-cycling microorganisms (Xu et al., 2020; Lin et al., 2021). Xin et al. found that a switch from inorganic N fertilization to organic N fertilization dramatically altered soil microbial community composition and co-occurrence networks. In addition, Wang et al. showed that long-term fertilization significantly influenced the transcriptions of N-cycling functional genes. Soil potential nitrification rate (PNR) was found to be significantly associated with the transcriptions of AOA *amoA* and *nxr* genes, while potential denitrification rate (PDR) was significantly correlated with *napA* and *nosZ* transcriptions. Long-term fertilization was observed to reduce PNR and enhance PDR mainly by regulating the transcriptions of N-cycling functional genes. Additionally, nutrient fertilization, particularly through animal manure application, can lead to an increasing co-contamination of heavy metals and antibiotics (Guo et al., 2018), which can also significantly influence N-cycling microorganisms. Cao et al. found that the contamination of oxytetracycline and/or cadmium inhibited soil net nitrification rates by mainly suppressing the growth of ammonia oxidizers.

The discovery of comammox *Nitrospira*, which can perform nitrification in a single cell, has revolutionized our understanding of nitrifying microorganisms (Daims et al., 2015; van Kessel et al., 2015). However, the niche differentiation of comammox *Nitrospira* and canonical ammonia oxidizers in terrestrial ecosystems remains unclear. Kits et al. (2017) revealed the oligotrophic lifestyle of comammox *Nitrospira* using kinetic analysis, whereas Li et al. (2023) suggested that comammox *Nitrospira* in terrestrial ecosystems may have a broader ecological niche breadth and not be strictly oligotrophic. Therefore, more evidence is needed to uncover the niche preference of comammox *Nitrospira* in soil ecosystems. Synthetic microbial ecology may be a promising approach for elucidating the niche differentiation of comammox *Nitrospira* and canonical ammonia oxidizers in the environment and how they respond to global change factors (Yang et al.). Unfortunately, comammox *Nitrospira* have not yet been isolated from soil environments until now, which constrains the development of synthetic microbial ecology approach in studying the characteristic of soil comammox *Nitrospira*.

To accurately assess the response and feedback of N-cycling microorganisms to global changes, the development of high-specificity and high-coverage primers for targeting these microorganisms is critical. Zhao et al. highlighted the importance of primer selection for prokaryotes and archaeal nitrifiers in soils, however, more efforts should be devoted to design better primers that can specifically target comammox *Nitrospira* in soils. This is particularly important, as currently available primers have been found to generate strong non-specific amplifications of comammox *Nitrospira amoA* genes (Pjevac et al., 2017; Lin et al., 2023).

Collectively, the articles published in this Research Topic offer novel perspectives on the response and feedback of N-cycling microorganisms to global environmental changes in terrestrial ecosystems, and emphasize the significance of developing effective methods and primers to study soil N-cycling microorganisms. We contend that global changes would exert a profound impact on N-cycling microorganisms and N cycle, highlighting the need for future research in this area.

## Author contributions

All authors listed have made a substantial, direct, and intellectual contribution to the work and approved it for publication.
